# Orthoform, a New Local Anesthetic

**Published:** 1898-11

**Authors:** 


### Orthoform, A New Local Anesthetic,

Does not substitute cocain, but has a field peculiar to itself. It is absolutely non-toxic; applied as a powder, it produces a slow, progressive anesthesia wherever there is solution in continuity, lasting hours and days. It has no effect upon sound skin or indurations, but in all burns, wounds, fissures, ulcerations, excoriations, etc., it abolishes sensibility, diminishes the secretions and exerts a pronounced antiseptic effect. Orthoform is a methyl ether compound of amidoxybenzoic acid, discovered by Drs. A. Einhorn and R. Heinz, of Munich. In combination with hydrochloric acid, it forms a soluble salt which can be administered internally, one-half to one gram several times a day, to remove the pain in cancer and round ulcer of the stomach. Intra-urethral injections in chronic gonorrhœa have also proved effectual.—*Semaine Med.*

